# The Enhanced Expression of ZWILCH Predicts Poor Survival of Adrenocortical Carcinoma Patients

**DOI:** 10.3390/biomedicines11041233

**Published:** 2023-04-21

**Authors:** Małgorzata Blatkiewicz, Kacper Kamiński, Marta Szyszka, Zaid Al-Shakarchi, Anna Olechnowicz, Ewelina Stelcer, Hanna Komarowska, Marianna Tyczewska, Anna Klimont, Marek Karczewski, Tomasz Wierzbicki, Joanna Mikołajczyk-Stecyna, Marek Ruchała, Ludwik K. Malendowicz, Marcin Ruciński

**Affiliations:** 1 Department of Histology and Embryology, Poznan University of Medical Sciences, 60-781 Poznan, Poland; 2 Doctoral School, Poznan University of Medical Sciences, 60-812 Poznan, Poland; 3 Department of Endocrinology, Metabolism and Internal Medicine, Poznan University of Medical Sciences, 60-356 Poznan, Poland; 4 Department of General and Transplantation Surgery, Poznan University of Medical Sciences, 60-356 Poznan, Poland; 5 Department of General, Endocrinological and Gastroenterological Surgery, Poznan University of Medical Sciences, 60-355 Poznan, Poland; 6 Department of Human Nutrition and Dietetics, Poznan University of Life Sciences, 60-624 Poznan, Poland

**Keywords:** adrenocortical carcinoma, adenoma, genetics, adrenal, *ZWILCH* gene

## Abstract

Zwilch kinetochore protein (ZWILCH) plays a key role in proper cell proliferation. The upregulation of the *ZWILCH* gene was observed in many types of cancers, but the association of ZWILCH with adrenocortical carcinoma (ACC) was not investigated so far. The main aim of the presented study was to verify if the enhanced level of the *ZWILCH* gene can be used as a diagnostic marker for ACC development and progression, as well as a predictor of survival time for ACC patients. The performed analyses included investigation of the *ZWILCH* expression profile in tumors with publicly available TCGA (The Cancer Genome Atlas) datasets and transcriptomic data from the Gene Expression Omnibus (GEO) database, as well as, in human biological samples of normal adrenal, adrenocortical carcinoma and in commercially available tissue microarrays. The findings demonstrate statistically significant higher *ZWILCH* gene expression in ACC tissue in comparison with normal adrenal glands. Furthermore, there is a strong correlation between *ZWILCH* upregulation and tumor mitotic rate and the probability of patient survival. The enhanced *ZWILCH* level is also connected with the activation of genes involved in cell proliferation and the inhibition of genes related to the immune system. This work contributes to a better understanding of the role of ZWILCH as an ACC biomarker and diagnostic tool.

## 1. Introduction

Adrenocortical carcinoma (ACC) is a severe and rare malignancy that affects approximately two individuals per million annually [1,2]. According to the WHO classification of adrenocortical tumors from 2017, the different subtypes of ACC include the main type, myxoid type, oncocytic type, sarcomatoid type, and pediatric type. [3]. Possible predictors for survival time among ACC patients are: Disease stage, defined by the European Network for the Study of Adrenal Tumours (ENSAT), radical surgery, age, cell proliferation potential, antigen Ki67, and Helsinki Score. The 5-year overall survival rate for subjects suffering from ACC is still low (15–44%) [4] and surgical intervention remains the only choice for long-term survival among patients with localized ACC [5].

Clinical manifestations of ACC associated with the overproduction of adrenal hormones, such as Cushing syndrome and hyperandrogenism are observed in approximately 50–70% of adult cases. The local symptoms resulting from tumor mass compression are found in about 30–40% of patients. Approximately 15% of ACC cases are diagnosed in a previously unsuspected adrenal mass discovered on chest or abdomen imaging examination [6]. Complete surgical resection remains the only curative ACC treatment. In patients with inoperable or metastatic ACC, mitotane is recommended as adjuvant therapy or treatment. In chemotherapy ACC patients with rapid disease progression, a combination of etoposide, doxorubicin, and cisplatin with mitotane is used [6].

Improved understanding of the molecular background of ACCs led to the identification of potential therapeutic and prognostic markers for these tumors [2]. The primary suggested oncogene involved in the development of ACC was insulin-like growth factor 2 (IGF2). Although the expression of *IGF2* is low or absent during the initial stages of clonal proliferation, upregulation of this gene has been demonstrated in over 85% of ACC cases [7]. Moreover, the activation of the Wingless-INT/Catenin Beta 1 (Wnt/CTNNB1) pathway correlates with a high mitotic rate and poor survival prognosis. Somatic mutations or loss of heterozygosity of gene coding Tumor Protein p53 (*TP53*) result in aberrant P53 expression and are linked to an aggressive phenotype, with a higher tumor stage and poorer survival rate [8]. Finally, Ki-67 may serve as the most important single factor in recurrence prediction and should be part of any ACC diagnosis to assess its proliferative potential [9,10]. Shortly, other non-invasive biomarkers for ACC should be also considered: microRNAs (miR-483-5p, miR-195, and miR-210); circulating tumor cells, and circulating cell-free tumor DNA which is a liquid biopsy-based approach [11].

Many human malignancies are characterized by chromosomal instability resulting from abnormal mitotic checkpoint function [12,13]. The Rod-Zw10-Zwilch complex, of which ZWILCH is an important component, plays a key role in the proper function of mitotic checkpoint [14]. The enhanced expression of *ZWILCH* was detected in many types of cancers: inter alia lung squamous carcinoma [15], colon cancer [12], hepatocellular carcinoma [16], and pancreatic cancer [17].

Despite ZWILCH expression being investigated in the mentioned types of cancer, its expression has not been studied yet in ACC [18]. The main goal of the present study was thus to determine the utility of *ZWILCH* gene expression, at the transcript and protein level, as a prognostic marker for ACC development and/or predictive factor in ACC patients. We hypothesize that there is a strong relationship between high malignancy and shorter life expectancy in patients with enhanced expression of *ZWILCH*. To verify our hypothesis, we performed a wide range of analyses: investigation of the *ZWILCH* expression profile in tumors with publicly available TCGA datasets (i), analysis of *ZWILCH* expression with publicly available transcriptomic data from the GEO database (ii), the study of *ZWILCH* expression in normal adrenals and adrenocortical carcinoma using biological samples (iii), and examination of ZWILCH protein expression in commercially available tissue microarrays, containing adrenal tumors (adrenal cancer progression) and normal adrenal gland tissues regarding Ki67 status and tumor size (iv). This research may explain the function of ZWILCH in ACC development and progression.

## 2. Materials and Methods

### 2.1. ZWILCH Gene Expression Profile in Adrenocortical Carcinoma (TCGA Database)

The analysis involved transcriptomic data from 92 adrenocortical carcinoma patients. Clinical data and gene expression normalized counts (transcript per million, TPM) were obtained from the TCGA publically available database using the FireBrowse server (http://gdac.broadinstitute.org/, accessed on 1 December 2022) [19]. The log2 transformation of TPM+1 values was applied to the extracted *ZWILCH* expression data. Time of survival and death events were subtracted from clinical data files and merged with *ZWILCH* expression. An overall survival area plot was generated for different estimates of the causal effect of the *ZWILCH* expression on survival using different Cox proportional hazards regression models. A contour plot was also generated with the probability of patient survival over time according to *ZWILCH* gene expression level in ACC patients. This analysis was performed using the “contsurvplot” library [20].

To subdivide the expression of the *ZWILCH* gene into separate categories, data from clinical data files concerning pathologic stages, pathologic categories (t), clinical categories (m), primary therapy outcome success, and archived therapeutic mitotane levels were used. Statistical differences in ZWILCH gene expression for comparisons with more than two categories were evaluated using the Kruskal–Wallis test followed by the Dunn post-hoc test. For comparisons with two categories, statistical differences were determined by the Mann–Whitney U test. A correlation analysis of *ZWILCH* gene expression with the gene encoding the KI67- *MKI67* was also performed. For this purpose, data for the *MKI67* gene were extracted and linear correlation was carried out using the Pearson correlation coefficient approach.

### 2.2. ZWILCH Gene Expression Profile in Normal Adrenal Cortex, Adrenocortical Adenomas Adrenocortical Carcinoma (Gene Expression Omnibus (GEO) Repository)

The analysis of transcriptomic data was obtained by Affymetrix HG U133 microarray plus 2.0 array (Affymetrix, Santa Clara, CA, USA), deposited in the publicly available GEO database (https://www.ncbi.nlm.nih.gov/geo/; accession number: GSE10927, accessed on 1 December 2022) [21]. Microarray CEL files from 10 normal adrenal cortex samples, 22 adrenocortical adenomas, and 33 adrenocortical carcinomas were downloaded into the R programming environment using “GEOquery” library [22]. The linear models for microarray data included in the “limma” library were utilized to determine the differential expression and statistical significance [23]. From the entire expression dataset, the data for *ZWILCH* gene expression was extracted and visualized as a boxplot with relevant statistics.

Further analyses focused only on the data of adrenocortical carcinomas with *ZWILCH* gene expression values referred to prognostic parameters from the clinical data file. In this context, the expression level of the *ZWILCH* gene was examined in groups with low and high Weiss scores. Subsequently, Pearson correlation of the *ZWILCH* gene with the mitotic rate of the tumor was carried out.

### 2.3. Co-Expression Analysis of the ZWILCH Gene with Other Genes from the Adrenocortical Carcinoma Transcriptome Profiles (GEO Repository)

From a group of 33 transcriptomic profiles of adrenocortical carcinomas, ten samples with the highest expression (*ZWILCH* up group) and ten with the lowest expression of the *ZWICH* gene (*ZWILCH* down group) were selected for further analysis. Differences in expression between the *ZWILCH* up and *ZWILCH* down groups with statistical evaluation were determined using linear models for microarray data implemented in the “limma” library. The obtained *p*-values were corrected using the FDR multiple-test correction method. Genes with fold change values greater than 1.5 or less than −1.5 and an adjusted *p*-value ≤ 0.05 were considered differentially expressed genes (DEGs). These genes were shown as green (up-regulated) or red (down-regulated) dots on a volcano plot. The DEGs that were upregulated and downregulated were subjected to functional annotation and clustering separately using the bioinformatics tool DAVID (Database for Annotation, Visualization, and Integrated Discovery) [24]. The “RDAVIDWebService” library was used to upload the ENTREZ IDs of DEGs to DAVID [25]. The DEGs were then matched with appropriate GO terms and significantly enriched GO terms were selected from the BP DIRECT’s GO database. Ontological groups containing more than 5 DEGs and with a corrected *p* < 0.05 (after Benjamini–Hochberg correction) were visualized as bubble plots. The “ComplexHeatmap” library was used to visualize genes from the ten most significantly enriched ontological groups (with the lowest adjusted *p*-value) after being subjected to hierarchical clustering [26].

Gene Set Enrichment Analysis (GSEA) was conducted using the “clusterProfiler” library [27]. The purpose of the analysis was to determine the level of depletion or enrichment in GO terms by calculating a normalized enrichment score (NES) with a respective *p*-value. The normalized fold change values of all genes were log2 transformed, sorted, and used as arguments for the “gseGO” function. Enrichment of gene sets was performed for the GO category “biological process,” assuming that the minimum size of each gene set for analysis = 50 and *p*-value cutoff = 0.05. Hierarchical clustering of enriched terms was then performed based on pairwise similarity calculations using the Jaccard similarity index. The result of the analysis classified individual GO terms into clusters based on their functional similarity. The resulting clusters were presented in the form of a tree diagram. The ten ontology groups with the highest enrichment score (highest NES value) and the ten groups with the most depleted enrichment score (lowest NES value) were visualized as a bar chart. Enrichment charts for the five most enriched and depleted GO terms were also presented.

### 2.4. Patients’ Characteristics

This study enrolled 14 patients who underwent adrenalectomy due to suspicion of ACC. Patient clinical data and tissue samples were obtained prior to adrenalectomy. For molecular analysis, the pathologically changed adrenals specimens (~0.5 cm^3^) were collected and preserved in RNAlater™ (#R0901, Sigma, St. Louis, MO, USA) for mRNA expression analysis or fixed in 10% buffered formalin for histological assessment. Unchanged adrenal gland samples from kidney donors were used as a control group (*n* = 6). The research protocol was accepted by the Local Ethics Committee of Poznan University of Medical Sciences (decision No. 31/22) and complied with the Declaration of Helsinki. The characteristics of the patients are presented in Table 1.

### 2.5. RNA Extraction and Quantification of Gene Expression

Total RNA from adrenal gland tissue was isolated using TRI reagent, with additional Dounce homogenization. Additionally, the RNA purification was performed using the Universal RNA Purification Kit (#E3599-02, EURx) according to the manufacturer’s protocol. The quantity of total mRNA was assessed using optical density at 260 nm and its purity was evaluated by the absorption ratio of 260/280 nm (greater than 1.8) using a NanoDrop spectrophotometer (ThermoFisher Scientific, Waltham, MA, USA). The cDNA synthesis was performed using iScript™ Select cDNA Synthesis Kit with Oligo(dT) (#1708897, Bio-Rad, Contra Costa County, CA, USA) (for each sample, 1 μg of total RNA was used) and stored at −20 °C. The obtained 20 µL of cDNA was suspended in 80 µL of nuclease-free water, resulting in a final 100 µL of cDNA, stored at −20 °C.

Quantitative real-time PCR was used to measure the expression of specific target genes, utilizing predesigned TaqMan™ Gene Expression Assays (ThermoFisher Scientific, Waltham, MA, USA) for human *ZWILCH* (Hs01555249_m1) and reference human *18S RNA* (Hs99999901_s1) and TaqMan™ Gene Expression Master Mix (#4370048, ThermoFisher Scientific, Waltham, MA, USA). Expression was measured by quantitative real-time PCR (CFX96, Bio-Rad) within 20 μL reaction mix (10 μL Master Mix, 1 μL assay, and 9 μL of cDNA template + Nuclease free water). Running the PCR reaction plate, the thermal cycling conditions were as follows: UNG incubation (2 min at 50 °C), AmpliTag Gold^®^, UP Enzyme activation (10 min at 95 °C), and 40 cycles of denaturation (15 s at 95 °C) and annealing (1 min at 60 °C). All samples were amplified in duplicate. The ΔΔCt quantification method was used to calculate the relative expression of the target genes.

### 2.6. The Tissue Microarray (TMA)

The tissue microarray slide was obtained as an unstained section of the adrenal gland disease spectrum (AD2081, US Biomax, Inc. Rockville, MD, USA) that included core samples of adrenal gland tissue contained 19 samples of adrenocortical carcinoma, 68 of adrenocortical adenoma, and 16 biopsy samples of normal adrenal tissue. Because commercially available TMA slides were used, this part of the study did not require the approval of the local bioethics committee.

### 2.7. Anti-ZWILCH Immunohistochemical (IHC) Staining

The detailed procedure for the preparation and staining of TMA sections was as previously described [28]. Briefly, after deparaffinization, the TMA section was rehydrated through a series of decreasing ethanol concentrations and subsequently washed in phosphate-buffered saline (PBS). The tissue samples were subjected to heat-induced epitope retrieval (HIER) processing to expose epitopes for the anti-ZWILCH antibodies. Slides were immersed in Target Retrieval Solution, Citrate pH 6.1 (#H-3300-250, Vector, Stuttgart, Germany), and heated in a microwave for 5 min. After that, they were cooled down to room temperature (RT) for 20 min. After endogenous peroxidase activity blocking and incubation with 2.5% normal horse serum, the sections were incubated with anti-ZWILCH polyclonal rabbit antibody (#14281-1-AP, Proteintech, Rosemont, IL, USA) with 1:1000 concentration at 4 °C overnight. Then, the tissue samples were washed and stained using ImmPRESS^®^ HRP Universal (Horse Anti-Mouse/Rabbit IgG) PLUS Polymer Kit (#MP-7800, Vector) according to the manufacturer’s instructions. The specimens were counterstained with Mayer’s hematoxylin (#S330930-2, DAKO, Glostrup, Denmark), followed by dehydration and mounting. The Mirax-Midi slide scanner (Zeiss, Jena, Germany) was used to digitize the whole slide. The IHC staining was analyzed and documented at a high magnification with Case-Viewer 2.3 (64-bit version) for Windows (3D Histech Ltd., Budapest, Hungary).

Semiquantitative analysis of ZWILCH protein expression was carried out by the densitometric method. Because our previous analyses involved normal adrenal glands, adenomas, and adrenocortical carcinomas, densitometric analysis was performed only for these groups. The blue–violet color consequent of hematoxylin staining was removed from the TMA image, retaining only the brown dye in the corresponding IHC reaction. The image was then converted to grayscale with color inversion. The full image preparation procedure was carried out using Adobe Photoshop ver. 21.1.0 (Adobe Inc., San Jose, CA, USA). After saving the resulting image in TIFF format, it was imported into the ImageJ software (ImageJ 1.5q, Wayne Rasband, National Institutes of Health, Bethesda, MD, USA), for densitometric analysis, which was performed according to The Open Lab Book protocol adapted to TMA format (the protocol is available at https://theolb.readthedocs.io/en/latest/imaging/measuring-cell-fluorescence-using-imagej.html, accessed on 1 December 2022). The integrated density was calculated from each of the TMA samples, with a fixed diameter covering 8800 pixels/piece. The measured pixel intensities for each tissue array core were calculated by taking into account the background signal.

The R programming language was used for all statistical analyses of densitometric values, with the “ggplot2” library utilized for visualization purposes. The densitometric values obtained from each of the studied groups were visualized as boxplots, indicating the median and interquartile range (IQR). The densitometric data for individual patients were overlaid on the correspoding boxplots and represented as dots. The Kruskal–Wallis test was used to compare the groups, followed by the Dunn post hoc test. The differences between groups were denoted using the letter annotation, where distinct letters indicate significant differences (*p* < 0.05). The *p*-value of the post hoc test for each pairwise comparison was also shown.

## 3. Results

### 3.1. High Expression of the ZWILCH Gene Reduces the Survival Probability of ACC Patients (Based on TCGA Data)

**Figure 1:**
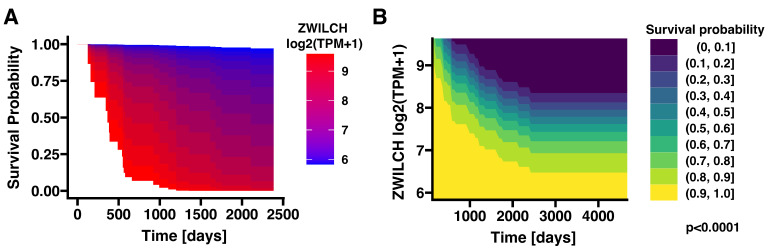
An overall survival area plot showing different estimates of the causal effect of the *ZWILCH* expression on survival using different Cox proportional hazards regression models (**A**). The contour plot illustrates the probability of patient survival over time according to *ZWILCH* gene expression level in ACC patients from the TCGA database (**B**).

The overall survival analysis of the *ZWILCH* expression profile in ACC tumors was performed on a publicly available TCGA dataset, indicating a strong negative correlation between the probability of survival and the expression of the *ZWILCH* gene. According to the survival analysis of continuous variables performed using the “contsurvplot” package, survival probability gradually decreases with increasing expression of the *ZWILCH* gene (Figure 1). Patients with a high expression of the *ZWILCH* gene have a shorter survival time and therefore a worse prognosis. Opposite, the survival probability of patients with a lower *ZWILCH* gene expression indicates a more promising prognosis.

**Figure 2:**
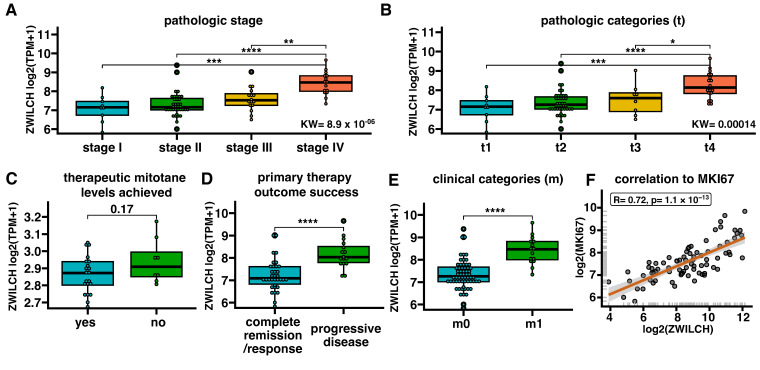
*ZWILCH* gene expression in ACC patients (data from TCGA) according to selected clinical characteristics of tumor progression (**A**–**E**). The bar plot presents the median with IQR range. Each dot represents *ZWILCH* expression in individual patients. Statistical differences in *ZWILCH* gene expression for comparisons with more than two categories were evaluated using the Kruskal–Wallis test followed by the Dunn post hoc test. For comparisons with two categories, statistical differences were determined by the Mann–Whitney U test. Correlation of *ZWILCH* and *KI67* expression in ACC patients (**F**). Differences between groups marked with asterisks: * *p* < 0.05, ** *p* < 0.01, *** *p* < 0.001, **** *p* < 0.0001. The graph presents the logarithmic values of TPM+1 for *ZWILCH* and *MKI67* genes. The regression line demonstrates a strong positive correlation (*p* = 1.1 × 10^−13^, R = 0.72). Each dot represents data from individual patients.

*ZWILCH* gene expression data were categorized according to clinical data (Figure 2). *ZWILCH* was found to increase with the progression of pathologic stages (I–IV) and pathologic categories (t1–t4), with a statistically significant difference for stages IV and t4. Although there was a trend of increased *ZWILCH* gene expression in patients who did not achieve therapeutic levels of mitotane, it did not reach statistical significance (Figure 2C). It is important to note that there were many missing values for this parameter in the clinical data files.

Significantly lower *ZWILCH* gene expression was observed in patients with complete remission/response (the primary outcome success, Figure 2D). Similarly, in terms of clinical categories (m), *ZWILCH* expression was found to be lower in m0 patients compared to m1 patients. Furthermore, a significantly positive correlation between the expression of *ZWILCH* and *KI67* in the cancer tissue of the patient (*p* = 1.1 × 10^−13^; R = 0.72) was observed (Figure 2F). The low expression of the *ZWILCH* gene is equal to the decreased expression of *KI67* in cancer tissue.

### 3.2. ZWILCH Expression Based on Gene Expression Omnibus Data

**Figure 3:**
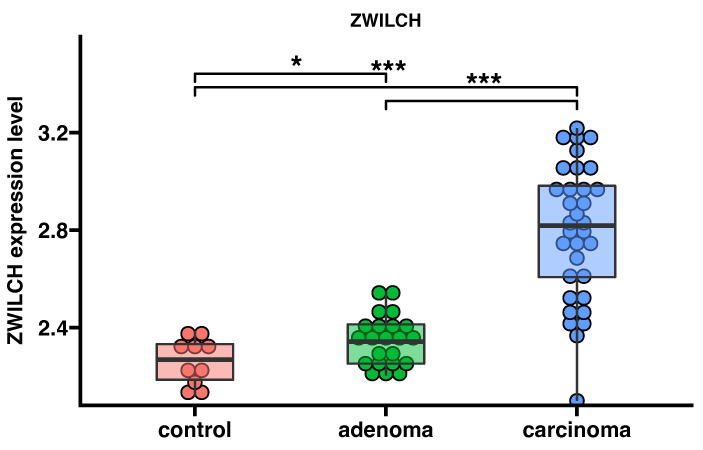
Expression profile of *ZWILCH* gene in the normal adrenal cortex (control, *n* = 10), adrenal adenoma (*n* = 22), and adrenocortical carcinoma (*n* = 33). Data were extracted from transcriptomic data deposited in the gene expression omnibus database (accession number: GSE10927). The bar plot presents the median with IQR range. Each dot represents *ZWILCH* expression in individual patients. Statistical differences were determined using the moderated *t* test * *p* < 0.05; *** *p* < 0.001.

The analysis of the publicly available transcriptomic data from the GEO database shows the expression of the *ZWILCH* gene in patients with adrenocortical adenomas (*n* = 22), adrenocortical carcinomas (*n* = 33), and control group (*n* = 10) (Figure 3). *ZWILCH* expression increased in both adrenocortical adenoma (*p* < 0.05) and adrenocortical carcinoma (*p* < 0.001) compared with the control group.

**Figure 4:**
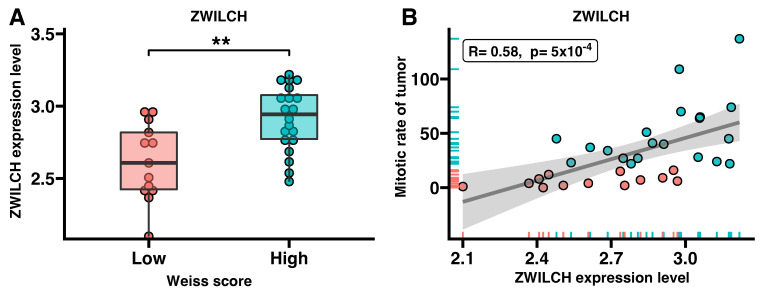
The expression of the *ZWILCH* gene in the group of patients with adrenocortical carcinoma according to Weiss score (Mann–Whitney U nonparametric test, ** *p <* 0.01) (**A**). Strong positive correlation of *ZWILCH* gene expression with the mitotic rate of tumor (R = 0.58, *p* = 0.0005) (**B**). The red dots represent patients with a low value of the Weiss score, while the turquoise dots represent a high value of the Weiss score.

Then, the *ZWILCH* expression data for the adrenocortical carcinoma were divided into high and low Weiss scores according to the value of the “Weiss grade of tumor” parameter provided in the clinical data file (Figure 4). We observed a significant increase in *ZWILCH* gene expression in patients with high Weiss scores (*p* < 0.01) (Figure 4A). Furthermore, there was a strong positive correlation between *ZWILCH* expression and tumor mitotic rate of (*p =* 0.0005, R = 0.58) (Figure 4B).

**Figure 5:**
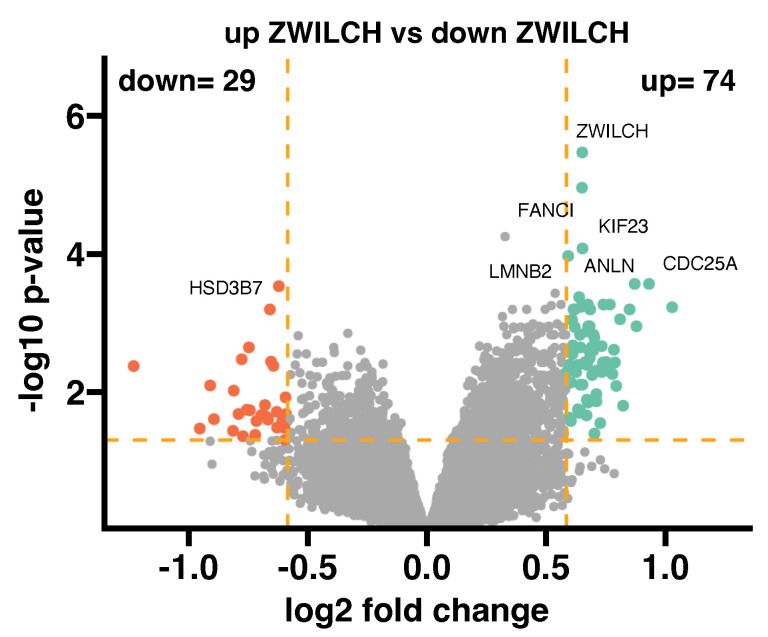
General transcriptome profile in groups of 10 patients with the highest expression of the *ZWILCH* gene (up *ZWILCH*) compared to 10 patients with the lowest expression of the *ZWILCH* gene (down *ZWILCH*) (data from GSE10927). Each dot on the graph represents the mean value of the gene expression level of ten patients belonging to a particular group. The cut-off criteria for identifying differentially expressed genes were established as |fold change| = 1.5 and *p*-value = 0.05, and are represented by orange dotted lines. Genes above the cut-off lines were categorized as up−regulated (green dots) or down−regulated (red dots). The total number of up− and down−regulated genes can be found in the top right and top left corners, respectively. The plots also indicate the symbols for the seven most differentially expressed genes.

To demonstrate the correlation of *ZWILCH* expression with other genes and relevant ontological groups, we collected transcriptomic data from ten adrenocortical carcinoma patients with the highest and the lowest *ZWILCH* expression. The transcriptome’s overall changes are displayed in Figure 5, where the mean value of gene expression is denoted by dots. Based on the preset cut-off criteria for identifying differentially expressed genes (|fold change| = 1.5 and *p*-value < 0.05), 74 genes were found to be upregulated, and 29 were downregulated in high-expressed *ZWILCH* probes when compared with low-expressed probes. The genes with the highest fold change of expression included Fanconi anemia, complementation group I (*FANCI*), Kinesin Family Member 23 (*KIF23*), Anillin (*ANLN*), Lamin B2 (*LMNB2*), and Cell Division Cycle 25A (*CDC25A*), while the mostly down-regulated genes were hydroxy-delta-5-steroid dehydrogenase, 3 beta- and steroid delta-isomerase 7 (*HSD3B7*). 

**Figure 6:**
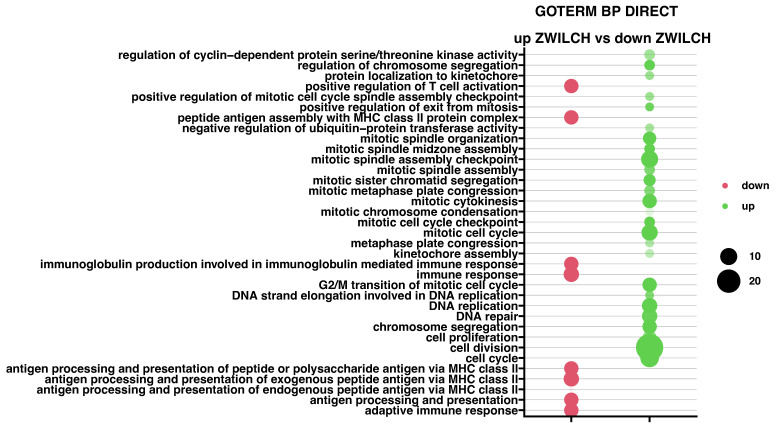
Bubble plot of enriched gene ontological terms by significantly up- or down-regulated genes from the whole transcriptome study, based on the DAVID GO PB DIRECT GO annotations database. The displayed graph presents only the GO groups above the established cut-off criteria (*p* with multiple testing correction <0.05, a minimal number of genes per group >5). The size of each bubble corresponds to the number of differentially expressed genes associated with the GO BP terms, while the degree of transparency reflects the *p*-value (more transparent indicates a value closer to the cut-off of *p* = 0.05). The green bubble indicates stimulated GO terms, while the red bubbles indicate the inhibited ones.

To verify the impact of *ZWILCH* expression on biological processes the DAVID GO PB DIRECT gene ontology (GO) annotation from the DAVID database was used (Figure 6). Comparison of groups with up- and down-regulated *ZWILCH* gene expression demonstrated 9 inhibited and 26 activated biological processes. It was noticed that all down-regulated processes are mainly related to immunological response and immune cell presentation. Meanwhile, upregulated processes are strictly related to cell proliferation and cell division. The highest statistical significance was shown for Inhibited process - “antigen processing and presentation of exogenous peptide antigen via MHC class II” (*n* = 8, *p with Benjamini correction* = 1.65 × 10^−13^) and activated process - “cell division” (*n* = 28, *p with Benjamini correction* = 1.65 × 10^−25^). 

**Figure 7:**
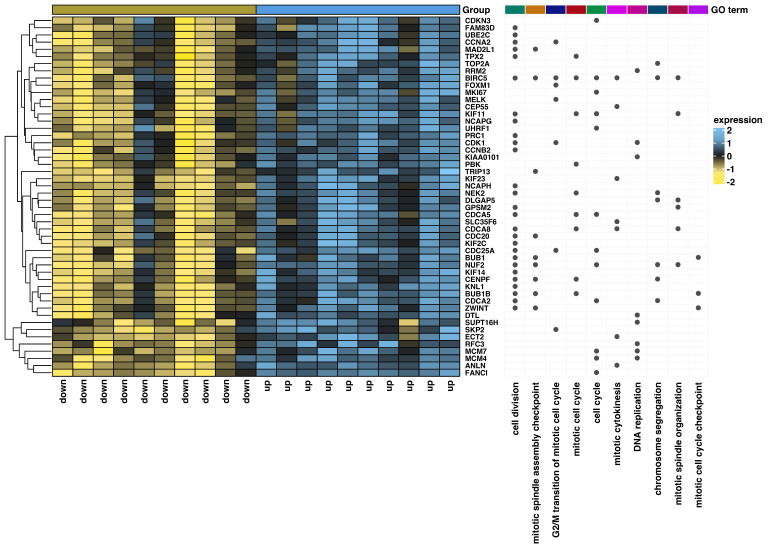
Heatmap of differentially expressed genes in the patients with adrenocortical carcinoma with the highest (up) and the lowest (down) expression of the *ZWILCH* gene. The heatmap on the right side displays the ten most significantly enriched ontological groups (with the lowest adjusted *p*−value), represented by dark dots. Expression values are scaled by rows and presented as color ranges from yellow (low expression) to blue (high expression).

**Figure 8:**
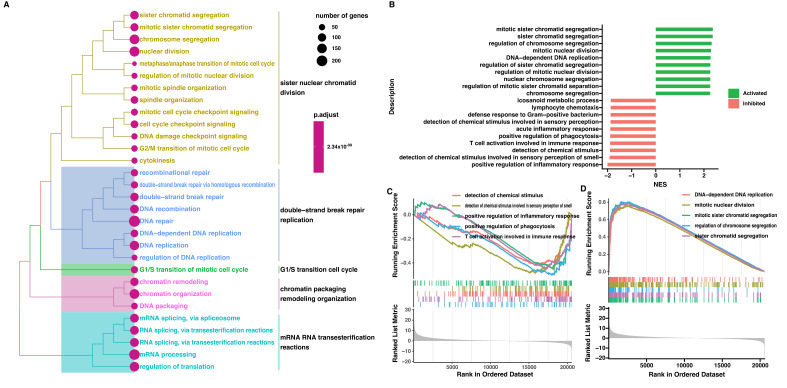
Gene set enrichment analysis (GSEA) from the comparison of patients with low and high *ZWILCH* expression, based on GSE10927. Clustering of enriched gene sets into common functional groups, each cluster is marked with a different color (**A**). The barplot with ten of the most activated and inhibited gene terms according to the normalized enrichment score values (**B**). Detailed enrichment plots for the five most inhibited and the five most activated gene sets, showing the profile of the running NES score and the positions of the genes on the rank−ordered list (**C**,**D**).

Furthermore, ten ontological terms with the lowest *p*-value were clustered and visualized on the heatmap (Figure 7). To confirm previously obtained results, we performed the GSEA (Figure 8). To generate the list of significantly represented terms, the normalized and ordered expression data from the microarray were uploaded to the clusterProfiler R package. The strongest enriched term in the comparison between patients with low and high *ZWILCH* expression refers to “mitotic sister chromatid segregation” (NES: 2.37), and “mitotic nuclear division” (NES: 2.29), which means that these processes were the most activated in patients with *ZWILCH* overexpression. Furthermore, the depletion (negative NES) enriched mainly genes involved in “positive regulation of inflammatory response” (NES: −2.00). Although utilizing a distinct methodological approach, the GSEA analysis demonstrates relatively similar results comparable to those presented in the analysis of ontological clusters using DAVID (Figure 8). 

### 3.3. ZWILCH Expression Adrenocortical Carcinoma Patient’s

**Figure 9:**
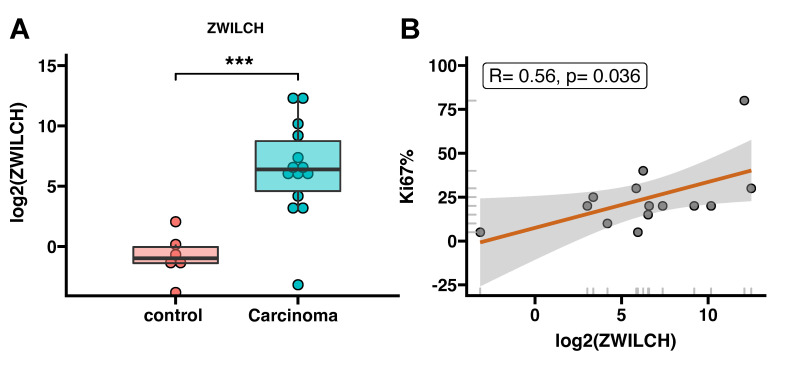
Adrenal expression of *ZWILCH* in patients with adrenocortical carcinoma (*n* = 14). Comparison of *ZWILCH* gene expression in ACC samples compared to normal adrenals–controls (*n* = 6). The bar plot presents the median with IQR range. Each dot represents *ZWILCH* expression in individual patients. Statistical differences were determined by the Mann−Whitney U nonparametric test *** *p* < 0.001 (**A**). Correlation analysis of *ZWILCH* expression and KI67 (**B**) in adrenocortical carcinoma patients. The graph shows the logarithmic value of *ZWILCH* gene expression and the percentage of KI67 (**B**). Linear regression demonstrates a strong positive correlation between *ZWILCH* expression and the percentage of KI67 (*p* = 0.036, R = 0.56) (**B**).

In further studies, we analyzed *ZWILCH* gene expression in normal adrenals and adrenocortical carcinoma using our own logical samples. In accordance with previously obtained results, we showed that *ZWIL*bio*CH* expression in adrenocortical carcinoma is significantly higher than in normal adrenal glands (5-fold increase, *p* < 0.001) (Figure 9A). Additionally, the expression of *ZWILCH* correlates with the KI67 marker (*p* = 0.036, R = 0.56), that also confirms our previous results (Figure 9B).

### 3.4. Protein Analisis of ZWILCH Expression and Localization

**Figure 10:**
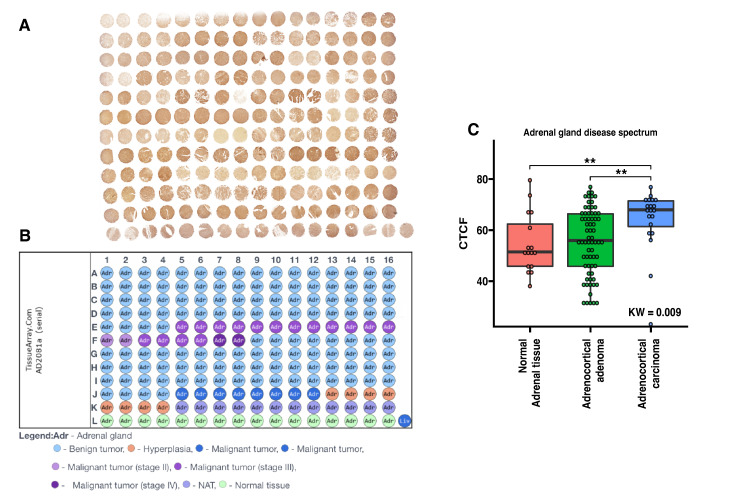
The expression of ZWILCH protein in human adrenal gland disease spectrum (adrenal cancer progression) tissue microarray (TMA) slide. The general profile of immunohistochemical staining presents localization of ZWILCH protein (**A**). TMA map shows the relevant types of adrenal cancer progression marked in an appropriate color (**B**). Densitometric analysis of ZWILCH gene expression in the tissue array side of the adrenal gland disease spectrum. The boxplot displays each group with its median and interquartile range (IQR) (**C**). Individual patient densitometric data were added to the corresponding boxplots and represented as dots. The Kruskal–Wallis (KW) test was used to compare groups, followed by the Dunn post hoc test. Differences between groups were marked with asterisks: ** for *p* < 0.01. Letter annotation was used to indicate statistically significant differences (*p* < 0.05) between compared groups. Paired comparisons with post hoc Dunn *p*-values are also shown in the table.

**Figure 11:**
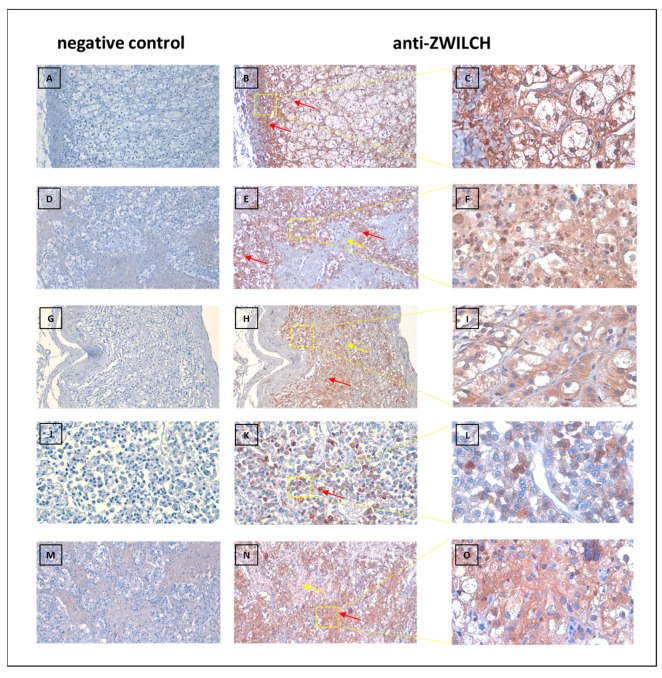
Representative immunostaining of adrenal gland disease spectrum (adrenal cancer progression) TMA slide. Brown staining (**B**,**C**,**E**,**F**,**H**,**I**,**K**,**L**,**N**,**O**) indicates ZWILCH protein (red arrows), located typically cellular with hematoxylin counterstain (nucleus). The negative control of adrenal gland tissue (**A**,**D**,**G**,**J**,**L**,**M**). Original magnification was 100× (**A**,**B**,**D**,**E**,**G**,**H**,**M**,**N**), 200× (**J**,**L**,**K**), and 400× (**C**,**F**,**I**,**L**,**O**).

To confirm the expression of the ZWILCH protein in tissues of the adrenal gland with different disease spectrum, immunohistochemical analyses were performed. The experiment involved analysis of ZWILCH protein expression with using of commercially available tissue microarrays, containing adrenal tumors (adrenal cancer progression) and normal adrenal gland tissues. The general profile of the stained specimens is illustrated in Figure 10. The immunohistochemical analysis showed an increased intensity of ZWILCH protein staining in adrenocortical carcinoma, thus confirming the results of our molecular analyses. The densitometric analysis of ZWILCH protein expression and Dunn *post hoc* test indicated significantly enhanced expression in adrenocortical carcinoma compared to the control group (*p*-value = 0.0053). However, no statistically significant differences were found in ZWILCH expression at the protein level between the control adrenal and adrenocortical adenoma tissues (*p*-value = 0.32). The analysis of protein localization indicated strong cytoplasmic expression of ZWILCH (Figure 11), but, in some cases, ZWILCH protein was found in the nucleus, which may be related to its role in the kinetochore function and depends on the stage of the cell cycle. Furthermore, we observed the highest ZWILCH expression level in the zona glomerulosa of the adrenal gland.

## 4. Discussion

In the presented study, we focused on the high-throughput analysis of *ZWILCH* expression in adrenocortical carcinoma. Our main goal was to evaluate the utility of *ZWILCH* as a potential marker of ACC development and/or predictive factor of disease prognosis.

Results obtained from the set of different analyses performed in our study (*ZWILCH* expression profile in tumors, performed on publicly available datasets (TCGA, GEO), confirmed by qPCR and IHC analysis) clearly and consistently showed the upregulation of *ZWILCH* in tumors compared with controls. It was also proven that the expression of *ZWILCH* is equal to the Ki67 expression in cancer tissue, and correlates with Weiss score and mitotic tumor rate. Moreover, we indicated enhanced expression of ZWILCH as an important factor of short survival time among adrenocortical carcinoma patients. 

As the association between ZWILCH and ACC has not been studied before, we discuss our findings in the context of the limited published data on the role of ZWILCH in different types of cancers.

ZWILCH, Rough-Deal (Rod), and Zeste-white 10 (Zw10) proteins are part of the RZZ complex, which plays a crucial role in the spindle assembly checkpoint. The entire complex’s construction is directly related to its function because the ZWILCH subunit is bound to the ROD β-propeller (WD40 domain). ROD’s α-solenoid region interacts with the centrally located ZW 10 subunit [29]. Therefore, the complex that is a component of the fibrillar crown of the kinetochore promotes the capture of microtubules [30]. Due to their function, the localization of the complex’s subunits changes during the cell cycle. During interphase, the subunits are primarily located in the cytoplasm. However, in later prophase and during nuclear envelope breakdown, they translocate to the nucleus and accumulate on kinetochores [31]. Moreover, to allow accurate chromosome segregation, the Polo kinase tightly regulates the RZZ–Spindly–dynein module. During mitosis, decreased Polo-kinase activity and Spindly dephosphorylation cause the RZZ susceptibility to removal from kinetochores by Spindly–dynein [32]. Thus, it has been shown that in the early prometaphase, inhibition of RZZ subunits and dynein/dynactin disruption results in transient poleward movement of chromosomes [33]. Despite numerous studies, the mechanism of RZZ’s complex remains to be fully understood. Our study demonstrated association between elevated expression of ZWILCH with upregulation of genes responsible for multiple stages of mitotic division, particularly mitotic sister chromatid segregation, checkpoints and cell cycle, therefore ZWILCH may play a major role in the regulation of cell proliferation. All these processes contribute to tumorigenesis and cancer progression. According to that, ZWILCH may indirectly be responsible for ACC development. On the other hand, observed association between *ZWILCH* upregulation and higher proliferation rate may be an effect of cell division rather than its origin by enhanced gene expression.

We noted also down-regulation of processes connected with the proper function of the immunological system within ZWILCH upregulation. Our results are consistent with already published lower levels of expression of genes involved in T-cell activation, which could be linked to tumorigenesis [34]. Zhang and collaborators (2021) have identified seven N6-methyladenosine-related immune prognostic genes (i.e., *PSMD10P1*, *DIDO1*, *ABCA5*, *CIITA*, *PRC1*,* ZWILCH*, and *ANLN*) for lung adenocarcinoma (LUAD) [35]. Noteworthy, high expression of *PRC1*, *ZWILCH*, and *ANLN* has been associated with low survival rates in LUAD patients, consistently with our results [35].

Furthermore, *ZWILCH* expression may be regulated by tumor suppressor gene products. Mizuno and co-workers (2021) have investigated the tumor-suppressive roles of *miR-150-3p* in lung squamous cell carcinoma (LUSQ) and its ability to control cancer-promoting genes in LUSQ cells [15]. The authors identified a total of 49 potential targets of miR-150-3p regulation in LUSQ cells, among which 17 genes, including *ZWILCH*, were classified under the “cell cycle” category based on GO classification [15]. Moreover, the lack of *miR-150-3p* as a direct control of the cell cycle regulator was correlated with the enhanced *ZWILCH* expression, which may be a starting point for further studies.

Chen and others (2020) have indicated *FANCI* and *ZWILCH* as crucial genes in colon cancer progression and proposed them as potential targets for colon cancer treatment [12]. Another study, which focused on the identification of 164 sorafenib resistance-related DEGs in hepatocellular carcinoma has revealed *ZWILCH* as important DEGs (*DYNLL2*, *H2AFJ*, *SHANK2*, *ZWILCH*, *CDC14A*, *IFT20*,* MTA3*, *SERPINA1*, and *TCF4*) involved in regulating multiple biological processes [16]. Multiple Yes-associated protein/TEA domain family member (YAP/TEAD)-regulated genes, including *ZWILCH* among other genes (*AJUBA*, *ANLN*, *AREG*, *ARHGAP29*, *AURKA*, *BUB1*, *CCND1*, *CDK6*, *CXCL5*, *ED N2*, *DKK1*, *FOSL1*, *FOXM1*, *HBEGF*, *IGFBP2*, *JAG1*, *NOTCH2*, *RHAMM*, *RRM2*, *SERP1*), have been linked to poor survival outcomes in individuals with pancreatic ductal adenocarcinoma [17].

Moreover, functional single-nucleotide polymorphisms (SNPs) were screened in both the regulatory and coding regions of six genes involved in different steps of mitosis that were correlated with chromosomal instability (CIN): *ZWILCH*. *CENPF*, *ESPL1*, *NEK2*, *PTTG1*, *ZWINT* [36]. The six SNPs were selected for subsequent genotyping analysis, but no significant differences were found in the allele or genotype frequencies between the breast cancer cases and the controls in relation to *ZWILCH* [36].

Hamam and colleagues (2014) have discovered several previously unknown gene targets of the *miR-320* family involved in the differentiation of human mesenchymal stem cells (hMSCs) into adipocytes [37]. The most relevant to adipogenesis were *MIB1*, *PAX6*, *YWHAH*, *ZWILCH*, and *RUNX2*. Small interfering RNA, RNA-mediated silencing of those genes, led to an increased number of adipocytes differentiated from hMSCs [37].

We are aware that our research has some limitations, the most important of which is the low number of patient samples used for validation data obtained from the bioinformatic analysis. Given that adrenocortical carcinoma is a rare disease, the group of patients we acquired is homogeneous in the context of clinical characteristics. Moreover, the results obtained from sample analyses indicate the role of ZWILCH in ACC even in a relatively small group of patients. However, a larger sample size and data collection are required to determine the role of ZWILCH and RZZ complexes in ACC conditions and will be considered in our future research plans.

To summarize, it should be emphasized that all the above-mentioned published data concerns the role of ZWILCH in different types of cancer, other than ACC. We showed for the first time such a comprehensive analysis of *ZWILCH* expression in the tissue of ACC patients. In conclusion, the use of *ZWILCH* to predict patient survival time would provide a valuable marker for diagnostic purposes. Our results extend also knowledge of the potential role of ZWILCH in adrenocortical carcinoma conditions. Consequently, this work fulfills the criteria of ”bench to bedside” research and may contribute to the development of personalized medicine.

## Figures and Tables

**Table 1:** Clinical characteristics of patients with ACC.

Age (y)	Mean (Min–Max.) 48.5 (26–71)
Sex (*n*)	Female 9; Male 5
Tumor size (mm)	Mean (min–max.) 136.4 (57–230)
Hormone secretion (*n*)	Glucocorticoids–3 Androgens–1 Glucocorticoids and androgens–5 Inactive–5
ENSAT tumor stage (*n*)	II–5 III–5 IV–4
Ki67index	Mean (min-max.) 26.25 (5–80)
BMI	Mean (min-max.) 25.32 (17.87–31.23)
Survival (months)	Mean (min-max.) 40.75 (3–116)
Deceased (*n*)	7

## Data Availability

Not applicable.

## References

[1] Vietor C.L., Creemers S.G., van Kemenade F.J., van Ginhoven T.M., Hofland L.J., Feelders R.A. (2021). How to Differentiate Benign from Malignant Adrenocortical Tumors?. Cancers.

[2] Ayala-Ramirez M., Jasim S., Feng L., Ejaz S., Deniz F., Busaidy N., Waguespack S.G., Naing A., Sircar K., Wood C.G. (2013). Adrenocortical carcinoma: Clinical outcomes and prognosis of 330 patients at a tertiary care center. Eur. J. Endocrinol..

[3] Minner S., Schreiner J., Saeger W. (2021). Adrenal cancer: Relevance of different grading systems and subtypes. Clin. Transl. Oncol..

[4] Kostiainen I., Hakaste L., Kejo P., Parviainen H., Laine T., Löyttyniemi E., Pennanen M., Arola J., Haglund C., Heiskanen I. (2019). Adrenocortical carcinoma: Presentation and outcome of a contemporary patient series. Endocrine.

[5] Kim Y., Margonis G.A., Prescott J.D., Tran T.B., Postlewait L.M., Maithel S.K., Wang T.S., Evans D.B., Hatzaras I., Shenoy R. (2016). Nomograms to Predict Recurrence-Free and Overall Survival After Curative Resection of Adrenocortical Carcinoma. JAMA Surg..

[6] De Filpo G., Mannelli M., Canu L. (2021). Adrenocortical carcinoma: Current treatment options. Curr. Opin. Oncol..

[7] Mizdrak M., Kurir T.T., Božić J. (2021). The Role of Biomarkers in Adrenocortical Carcinoma: A Review of Current Evidence and Future Perspectives. Biomedicines.

[8] Jouinot A., Bertherat J. (2018). Management of endocrine disease: Adrenocortical carcinoma: Differentiating the good from the poor prognosis tumors. Eur. J. Endocrinol..

[9] Zhang F., Zhang F., Liu Z., Wu K., Zhu Y., Lu Y. (2019). Prognostic Role of Ki-67 in Adrenocortical Carcinoma After Primary Resection: A Retrospective Mono-Institutional Study. Adv. Ther..

[10] Beuschlein F., Weigel J., Saeger W., Kroiss M., Wild V., Daffara F., Libe R., Ardito A., Al Ghuzlan A., Quinkler M. (2015). Major Prognostic Role of Ki67 in Localized Adrenocortical Carcinoma After Complete Resection. J. Clin. Endocrinol. Metab..

[11] Cheng Y., Kou W., Zhu D., Yu X., Zhu Y. (2022). Future Directions in Diagnosis, Prognosis and Disease Monitoring of Adrenocortical Carcinoma: Novel Non-Invasive Biomarkers. Front. Endocrinol..

[12] Chen W., Gao C., Liu Y., Wen Y., Hong X., Huang Z. (2020). Bioinformatics Analysis of Prognostic miRNA Signature and Potential Critical Genes in Colon Cancer. Front. Genet..

[13] Komatsu M., Yoshimaru T., Matsuo T., Kiyotani K., Miyoshi Y., Tanahashi T., Rokutan K., Yamaguchi R., Saito A., Imoto S. (2013). Molecular features of triple negative breast cancer cells by genome-wide gene expression profiling analysis. Int. J. Oncol..

[14] Kops G.J.P.L., Weaver B.A.A., Cleveland D. (2005). On the road to cancer: Aneuploidy and the mitotic checkpoint. Nat. Rev. Cancer.

[15] Mizuno K., Tanigawa K., Misono S., Suetsugu T., Sanada H., Uchida A., Kawano M., Machida K., Asai S., Moriya S. (2021). Regulation of Oncogenic Targets by Tumor-Suppressive *miR-150-3p* in Lung Squamous Cell Carcinoma. Biomedicines.

[16] Liu J., Qiu W., Shen X., Sun G. (2019). Bioinformatics analysis revealed hub genes and pathways involved in sorafenib resistance in hepatocellular carcinoma. Math. Biosci. Eng..

[17] Rozengurt E., Sinnett-Smith J., Eibl G. (2018). Yes-associated protein (YAP) in pancreatic cancer: At the epicenter of a targetable signaling network associated with patient survival. Signal Transduct. Target. Ther..

[18] Tian X., Xu W., Anwaier A., Wang H., Wan F., Cao D., Luo W., Shi G., Qu Y., Zhang H. (2021). Construction of a robust prognostic model for adult adrenocortical carcinoma: Results from bioinformatics and real-world data. J. Cell. Mol. Med..

[19] Deng M., Brägelmann J., Kryukov I., Agostinho N.D.S., Perner S. (2017). FirebrowseR: An R client to the Broad Institute’s Firehose Pipeline. Database.

[20] Denz R., Timmesfeld N. (2022). Visualizing the Causal Effect of a Continuous Variable on a Time-to-Event Outcome. arXiv.

[21] Giordano T.J., Kuick R., Else T., Gauger P.G., Vinco M., Bauersfeld J., Sanders D., Thomas D.G., Doherty G., Hammer G. (2009). Molecular Classification and Prognostication of Adrenocortical Tumors by Transcriptome Profiling. Clin. Cancer Res..

[22] Davis S., Meltzer P.S. (2007). GEOquery: A bridge between the Gene Expression Omnibus (GEO) and BioConductor. Bioinformatics.

[23] Ritchie M.E., Phipson B., Wu D., Hu Y., Law C.W., Shi W., Smyth G.K. (2015). limma powers differential expression analyses for RNA-sequencing and microarray studies. Nucleic Acids Res..

[24] Dennis G., Sherman B.T., Hosack D.A., Yang J., Gao W., Lane H.C., Lempicki R.A. (2003). DAVID: Database for Annotation, Visualization, and Integrated Discovery. Genome Biol..

[25] Fresno C., Fernández E.A. (2013). RDAVIDWebService: A versatile R interface to DAVID. Bioinformatics.

[26] Gu Z., Eils R., Schlesner M. (2016). Complex heatmaps reveal patterns and correlations in multidimensional genomic data. Bioinformatics.

[27] Yu G., Wang L.-G., Han Y., He Q.-Y. (2012). clusterProfiler: An R Package for Comparing Biological Themes Among Gene Clusters. OMICS J. Integr. Biol..

[28] Komarowska H., Malinska A., Komekbai Z., Brominska B., Bednarek-Rajewska K., Ruchala M., Rucinski M. (2021). Immunohistochemical analysis of ghrelin expression in various types of adrenal tumors. Folia Histochem. Cytobiol..

[29] Henen M.A., Myers W., Schmitt L.R., Wade K.J., Born A., Nichols P.J., Vögeli B. (2021). The Disordered Spindly C-terminus Interacts with RZZ Subunits ROD-1 and ZWL-1 in the Kinetochore through the Same Sites in C. Elegans. J. Mol. Biol..

[30] Menant A., Karess R.E. (2020). Mutations in the Drosophila rough deal gene affecting RZZ kinetochore function. Biol. Cell.

[31] Karess R. (2005). Rod-Zw10-Zwilch: A key player in the spindle checkpoint. Trends Cell Biol..

[32] Barbosa J.F., Martins T., Bange T., Tao L., Conde C., Sunkel C. (2020). Polo regulates Spindly to prevent premature stabilization of kinetochore–microtubule attachments. EMBO J..

[33] Gassmann R., Essex A., Hu J.S., Maddox P.S., Motegi F., Sugimoto A., O’Rourke S.M., Bowerman B., McLeod I., Yates J.R. (2008). A new mechanism controlling kinetochore-microtubule interactions revealed by comparison of two dynein-targeting components: SPDL-1 and the Rod/Zwilch/Zw10 complex. Genes Dev..

[34] Hanahan D., Weinberg R.A. (2011). Hallmarks of cancer: The next generation. Cell.

[35] Zhang L., Luo Y., Cheng T., Chen J., Yang H., Wen X., Jiang Z., Li H., Pan C. (2021). Development and Validation of a Prognostic N6-Methyladenosine-Related Immune Gene Signature for Lung Adenocarcinoma. Pharmacogenom. Pers. Med..

[36] Brendle A., Brandt A., Johansson R., Enquist K., Hallmans G., Hemminki K., Lenner P., Försti A. (2009). Single nucleotide polymorphisms in chromosomal instability genes and risk and clinical outcome of breast cancer: A Swedish prospective case-control study. Eur. J. Cancer.

[37] Hamam D., Ali D., Vishnubalaji R., Hamam R., Al-Nbaheen M., Chen L., Kassem M., Aldahmash A., Alajez N.M. (2014). microRNA-320/RUNX2 axis regulates adipocytic differentiation of human mesenchymal (skeletal) stem cells. Cell Death Dis..

